# Beneficial effect of 24-month bilateral subthalamic stimulation on quality of sleep in Parkinson’s disease

**DOI:** 10.1007/s00415-020-09743-1

**Published:** 2020-03-09

**Authors:** Haidar S. Dafsari, K. Ray-Chaudhuri, Keyoumars Ashkan, Lena Sachse, Picabo Mahlstedt, Monty Silverdale, Alexandra Rizos, Marian Strack, Stefanie T. Jost, Paul Reker, Michael Samuel, Veerle Visser-Vandewalle, Julian Evans, Angelo Antonini, Pablo Martinez-Martin, Lars Timmermann, EUROPAR, the IPMDS Non Motor P.D. Study Group, Kallol Ray-Chaudhuri, Angelo Antonini, Pablo Martinez-Martin, Anette Schrag, Daniel Weintraub, Paolo Barone, David J. Brooks, Richard G. Brown, Peter Jenner, B. Jeon, Kelly Lyons, Nicola Pavese, Marios Politis, Ronald B. Postuma, Anthony Schapira, Fabrizio Stocchi, Lars Timmermann, Yoshio Tsuboi, Alexandra Rizos, Anna Sauerbier

**Affiliations:** 1 Department of Neurology, University Hospital Cologne, Kerpenerstr. 62, 50937 Cologne, Germany; 2 National Parkinson Foundation International Centre of Excellence, King’s College Hospital, London, UK; 3 Institute of Psychiatry, Psychology and Neuroscience, King’s College London, London, UK; 4 Department of Neurology and Neurosurgery, Salford Royal NHS Foundation Trust, Manchester Academic Health Science Centre, University of Manchester, Greater Manchester, UK; 5 Department of Stereotaxy and Functional Neurosurgery, University Hospital Cologne, Cologne, Germany; 6 Parkinson and Movement Disorders Unit, IRCCS Hospital San Camillo, Venice, Italy; 7 Department of Neurosciences (DNS), Padova University, Padova, Italy; 8 National Center of Epidemiology and CIBERNED, Carlos III Institute of Health, Madrid, Spain; 9 Department of Neurology, University Hospital Giessen and Marburg, Campus Marburg, Marburg, Germany

**Keywords:** Deep brain stimulation, Subthalamic nucleus, Non-motor symptoms, Quality of life, Parkinson’s disease sleep scale

## Abstract

**Background:**

Subthalamic nucleus (STN) deep brain stimulation (DBS) improves quality of life (QoL), motor, and sleep symptoms in Parkinson’s disease (PD). However, the long-term effects of STN-DBS on sleep and its relationship with QoL outcome are unclear.

**Methods:**

In this prospective, observational, multicenter study including 73 PD patients undergoing bilateral STN-DBS, we examined PDSleep Scale (PDSS), PDQuestionnaire-8 (PDQ-8), Scales for Outcomes in PD-motor examination, -activities of daily living, and -complications (SCOPA-A, -B, -C), and levodopa-equivalent daily dose (LEDD) preoperatively, at 5 and 24 months follow-up. Longitudinal changes were analyzed with Friedman-tests or repeated-measures ANOVA, when parametric tests were applicable, and Bonferroni-correction for multiple comparisons. Post-hoc, visits were compared with Wilcoxon signed-rank/*t*-tests. The magnitude of clinical responses was investigated using effect size.

**Results:**

Significant beneficial effects of STN-DBS were observed for PDSS, PDQ-8, SCOPA-A, -B, and -C. All outcomes improved significantly at 5 months with subsequent decrements in gains at 24 months follow-up which were significant for PDSS, PDQ-8, and SCOPA-B. Comparing baseline and 24 months follow-up, we observed significant improvements of PDSS (small effect), SCOPA-A (moderate effect), -C, and LEDD (large effects). PDSS and PDQ-8 improvements correlated significantly at 5 and 24 months follow-up.

**Conclusions:**

In this multicenter study with a 24 months follow-up, we report significant sustained improvements after bilateral STN-DBS using a PD-specific sleep scale and a significant relationship between sleep and QoL improvements. This highlights the importance of sleep in holistic assessments of DBS outcomes.

## Introduction

Subthalamic nucleus (STN) deep brain stimulation (DBS) is a safe and effective treatment option improving quality of life (QoL) [18], motor [28], and non-motor symptoms (NMS) [14] in patients with advanced Parkinson’s disease (PD) who suffer from motor complications or pharmacotherapy-refractory tremor [46].

In patients with PD, sleep symptoms are common and associated with QoL impairments [44]. Previous studies, using PD-specific clinician-rated scales [14] and patient-based self-reported questionnaires [4, 25, 37, 38], have provided evidence for beneficial effects of STN-DBS on sleep symptoms [38]. More recently, a study by Choi et al. found significant sustained improvements in sleep disturbances up to 3 years after STN-DBS [6]. But the study was limited by the single center design of their study, the cohort sample size (45 patients completed the last follow-up, and the mean disease duration of patients (17.2 years ± 6.2), which is longer than most DBS studies [12, 18, 49, 51].

These results were supported by studies using polysomnography which showed an improvement of sleep architecture [1, 7, 35], time of wakefulness after sleep onset [1, 7, 37], time of REM sleep time [35, 37], and periodic limb movements [3]. However, long-term beneficial effects on sleep symptoms and if these relate to an improvement of QoL after STN-DBS have not been studied sufficiently. A study by Lyons et al. reported negative results for daytime sleepiness at 24 months follow-up after STN-DBS but did not investigate overall quality of sleep and nocturnal sleep symptoms [30].

Here we report subjective sleep symptoms at 5 months and 24 months follow-up in patients with PD undergoing STN-DBS. We hypothesized that sleep symptoms significantly improve from baseline to 24 months follow-up and that this beneficial effect is significantly correlated with an improvement of QoL after STN-DBS.

## Materials and methods

### Design and ethical approval

This is an ongoing, prospective, observational, multicenter, international study including consecutively enrolled patients from three DBS centers (Cologne, Manchester, and London) as part of the NILS study [9]. It was authorized by local ethics committees (United Kingdom: NRES SouthEast London REC3, 0000010084; 10/H0808/141; Cologne 012–145, German Clinical Trials Register: #6735) and was carried out in accordance with the Declaration of Helsinki. All patients gave written consent prior to study procedures.

### Participants

PD diagnosis was based on the UK Brain Bank criteria and DBS screening was carried out according to guidelines of the International PD and Movement Disorders Society. Patients were considered eligible for DBS treatment if the levodopa test resulted in > 30% improvement of motor examination assessed by the Unified PD Rating Scale-III. Patients were excluded from DBS treatment if clinically relevant neuropsychological or neuropsychiatric disorders were found in assessments by a multi-disciplinary team including specialized neuropsychiatrists and neuropsychologists.

### Clinical assessment

Patients were assessed at baseline (MedON) and at 5 and 24 months follow-up visits after surgery (MedON/StimON) with following scales:

1. Sleep symptoms: the patient-based self-reported PD Sleep Scale (PDSS) was employed to investigate fifteen disease-specific aspects of sleep rated on a visual analog scale (item 1: ‘Overall sleep quality’, item 2: ‘Sleep onset insomnia’, item 3: ‘Sleep maintenance insomnia’, item 4 ‘Nocturnal restlessness in legs or arms’, item 5: ‘Fidgeting in bed’, item 6: ‘Distressing dreams at night’, item 7: ‘Distressing hallucinations at night’, item 8: ‘Nocturia’, item 9: ‘Urinary incontinence due to motor OFF’, item 10: ‘Wakefulness due to numbness/tingling’, item 11: ‘Wakefulness due to painful muscle cramps’, item 12: ‘Early waking due to painful posturing’, item 13: ‘Tremor on wake up’, item 14: ‘Sleep refreshment’, item 15: ‘Unexpectedly falling asleep at daytime’). The clinimetric properties of the overall PDSS and its specific items and their strong relationship with other sleep–wake disorder scores (e.g. the strong correlation between PDSS item 15 and the Epsworth Sleepiness Scale) have been well established [5, 50]. PDSS items respectively range from 0 (maximum impairment) to 10 (no impairment). Therefore, the PDSS total score ranges from 0 (maximum impairment) to 150 (no impairment).
2. QoL: the PD Questionaire-8 (PDQ-8) has previously been used in patients with PD and STN-DBS [13, 47]. The PDQ is recommended for assessments of QoL by the Movement Disorders Society Scales Committee [33] and commonly used for DBS studies in PD [18, 45]. Results are reported as PDQ-8 Summary Index (PDQ-8 SI) to help the interpretation of results and simplify comparisons with other studies. The PDQ-8 SI ranges from 0 (no impairment) to 100 (maximum impairment).
3. Mood disorder: the Hospital Anxiety and Depression Scale subscales for anxiety and depression (HADS-A and -D) was used to examine specific mood disorders [11, 42]. The HADS-A and -D subscale range from 0 (no anxiety/depression) to 21 (maximum anxiety/depression).
4. Motor disorder: the Scales for Outcomes in PD (SCOPA)-A, -B, and -C were used to assess respectively motor examination, activities of daily living, and motor complications. The SCOPA is an abbreviated version of the Unified PD Rating Scale from which it was derived [31] and the two scales highly correlate [32]. The SCOPA-A, -B, and -C range from 0 (no impairment) to 42, 21, and 12 respectively (maximum impairment).
5. The therapeutic medical regimen was recorded calculating the total levodopa equivalent daily dose (LEDD) and the LEDD of dopamine agonists according to the method of Tomlinson et al. [49].

### Statistical analysis

Normality of distribution of clinical scores was tested with the Shapiro–Wilk method. Significant longitudinal changes of outcome parameters were analyzed with Friedman-tests or repeated-measures ANOVA, when parametric tests were applicable. As we used multiple tests, the Bonferroni-correction for multiple comparisons was applied. The already corrected *p*-values are presented here (significance threshold: *p* = 0.05). Post-hoc Wilcoxon signed-rank t-tests, respectively were employed to investigate significant changes between the three visits. To investigate the magnitude of changes, we calculated effect sizes ([mean Test_visit 1_ − mean Test_visit 2_]/SD Test_visit 1_) [8] and relative changes ([mean Test_visit 2_ − mean Test_visit 1_]/mean Test_visit 2_).

Furthermore, we investigated the relationship between changes of all outcome parameters at 24 months follow-up by computing Spearman correlations between change scores (Test_change scores_ = Test_baseline_ − Test_follow-up_). We also explored Spearman correlations for change scores from 5 to 24 months follow-up for LEDD (total and dopamine agonist) and PDSS (total score and items).

## Results

The study included 73 patients (47 males) with PD undergoing bilateral STN-DBS. Patients were aged 61.9 years ± 7.7 with 10.4 years ± 5.0 disease duration. The median Hoehn and Yahr was 2.5 (interquartile range: 2.0–3.0).

### Clinical outcomes at baseline, 5 months, and 24 months follow-up

**Fig. 1:**
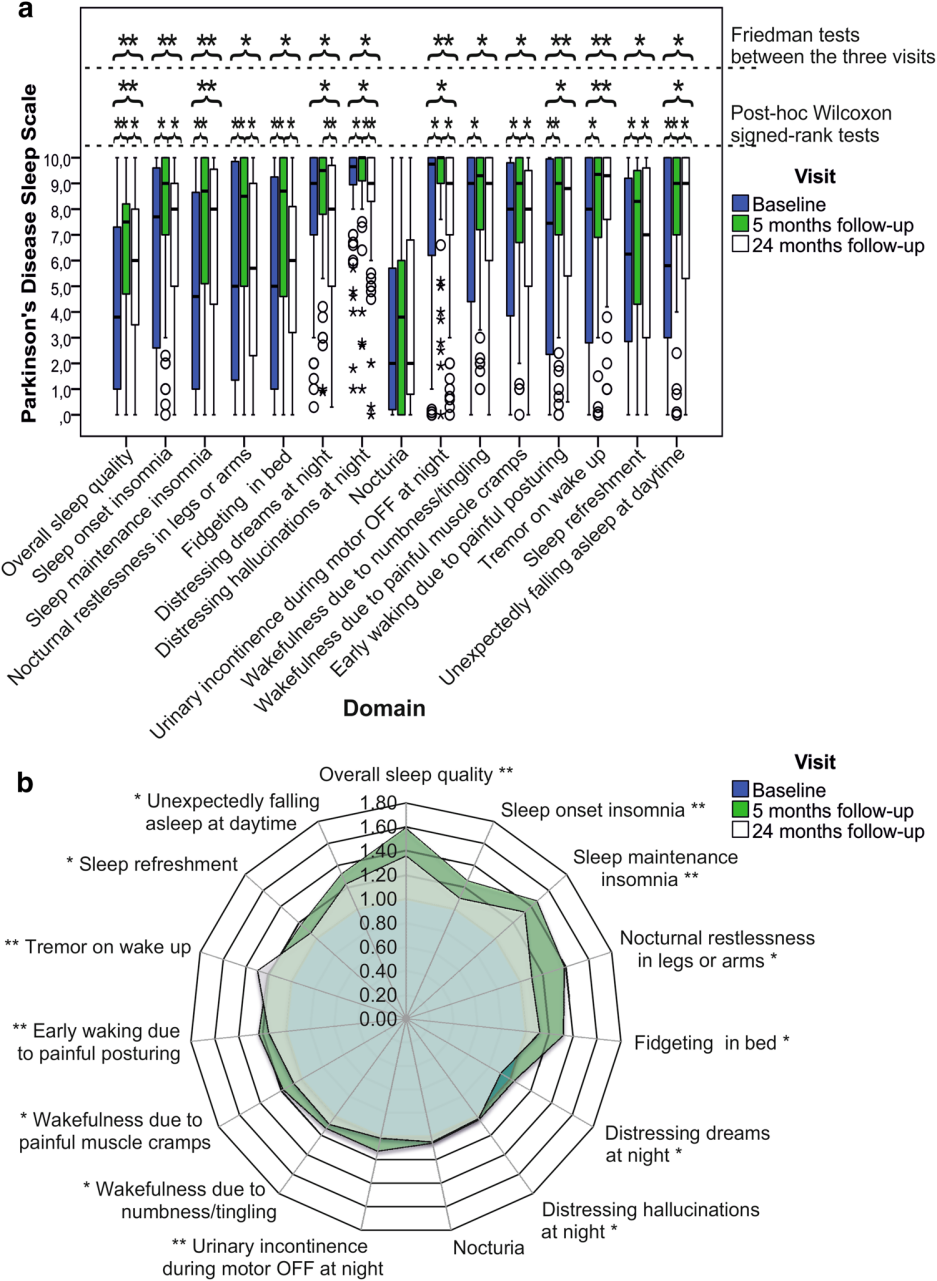
Parkinson’s Disease Sleep Scale at preoperative baseline and postoperative follow-up at 5 and 24 months

Friedman-tests, repeated-measures ANOVA resulted in significant longitudinal changes of all outcome parameters (see Table 1 and Fig. 1). Comparing baseline to 5 months follow-up, post-hoc Wilcoxon signed-rank, *t*-tests found significant improvements of all outcome parameters (all *p* < 0.001) with subsequent decrements in these gains from 5 to 24 months follow-up. This decrement reached statistical significance for PDSS total score, PDQ-8 SI, HADS-D (all *p* < 0.001), HADS-A (*p* = 0.011), and SCOPA-B (*p* = 0.011). Nonetheless, comparing baseline to 24 months follow-up, significant beneficial effects of bilateral STN-DBS were observed for PDSS total score, SCOPA-A, -B, -C, total LEDD, and dopamine agonists LEDD (SCOPA-B *p* = 0.046, all other *p* < 0.001).

**Table 1:** Outcome parameters at baseline, 5 months and 24 months follow-up

	*n*	Baseline		5 Months follow-up		24 Months follow-up		*p*^a^	Post-hoc tests^b^
		Mean	SD	Mean	SD	Mean	SD		
PDSS total score **	66	90.0	25.3	111.1	22.9	98.9	22.1	**< 0.001**	a^ǂ^, b^ǂ^, c^ǂ^
PDSS item 1: ‘overall sleep quality’ **	67	4.2	3.1	6.5	2.6	5.7	2.8	**< 0.001**	a^ǂ^, b^ǂ^, c
PDSS item 2: ‘sleep onset insomnia’ **	68	6.3	3.6	7.8	2.8	7.0	2.9	**0.001**	a, c
PDSS item 3: ‘sleep maintenance insomnia’ **	66	4.8	3.7	7.2	3.1	6.7	3.0	**< 0.001**	a^ǂ^, b^ǂ^
PDSS item 4: ‘nocturnal restlessness in legs or arms’ *	68	5.0	3.8	6.9	3.4	5.4	3.3	**0.002**	a^ǂ^, c
PDSS item 5: ‘fidgeting in bed’ *	68	5.2	3.8	6.8	3.4	5.8	3.1	**0.026**	a^ǂ^, c
PDSS item 6: ‘distressing dreams at night’ *	67	7.9	2.8	8.2	2.7	7.3	2.7	**0.027**	b, c^ǂ^
PDSS item 7: ‘distressing hallucinations at night’ *	68	8.8	2.0	9.3	1.8	8.4	2.4	**0.002**	a, c^ǂ^
PDSS item 8: ‘nocturia’	67	3.4	3.5	3.5	3.3	3.4	3.3	0.497	
PDSS item 9: ‘urinary incontinence during motor OFF’ **	68	7.8	3.3	8.7	2.4	7.7	3.0	**0.001**	a, c
PDSS item 10: ‘wakefulness due to numbness/tingling’ *	68	6.9	3.4	7.9	2.9	7.7	2.8	**0.049**	a
PDSS item 11: ‘wakefulness due to painful muscle cramps’ *	67	6.5	3.3	7.7	2.8	7.1	3.0	**0.005**	a, c
PDSS item 12: ‘early waking due to painful posturing’ **	68	6.3	3.7	7.7	3.0	7.3	3.1	**< 0.001**	a^ǂ^, b
PDSS item 13: ‘tremor on wake up’ **	66	6.2	3.8	7.8	3.2	8.3	2.3	**< 0.001**	a, b^ǂ^
PDSS item 14: ‘sleep refreshment’ *	68	5.8	3.5	6.8	3.2	6.2	3.4	**0.014**	a, c
PDSS item 15: ‘unexpectedly falling asleep at daytime’ *	67	5.9	3.7	7.8	3.0	7.3	3.0	**0.004**	a^ǂ^, b, c
PDQ-8 SI **	72	33.1	17.1	22.7	14.1	30.1	18.7	**< 0.001**	a^ǂ^, c^ǂ^
HADS-A	70	5.9	3.7	4.6	3.3	5.4	4.1	**0.002**	a^ǂ^, c
HADS-D	70	4.9	3.0	3.9	2.9	5.2	3.2	**0.006**	a, c^ǂ^
SCOPA-A **	67	12.6	6.0	8.6	4.9	9.1	5.0	**< 0.001**	a^ǂ^, b^ǂ^
SCOPA-B **	71	7.7	3.6	5.6	2.8	6.7	3.8	**< 0.001**	a^ǂ^, b, c
SCOPA-C **	71	5.3	3.1	2.8	2.6	2.9	2.4	**< 0.001**	a^ǂ^, b^ǂ^
LEDD total (mg) **	69	1103.8	503.4	641.4	365.1	702.3	442.0	**< 0.001**	a^ǂ^, b^ǂ^
LEDD dopamine agonists (mg)**	69	293.0	245.4	153.9	139.3	133.4	116.2	**< 0.001**	a^ǂ^, b^ǂ^

Bold font highlights significant results

*HADS-A and -D *Hospital Anxiety and Depression Scale-anxiety and -depression subscales, *LEDD* levodopa equivalent daily dose, *PDSS* Parkinson’s Disease Sleep Scale, *PDQ-8 SI* 8-item Parkinson’s Disease Questionnaire Summary Index, *SCOPA-A, -B, -C* Scales for Outcomes in Parkinson’s disease-motor examination, -activities of daily living, -motor complications

Post-hoc comparisons (Wilcoxon signed-rank or *t*-tests):

Baseline vs 5 months follow-up: a = significant (*p* < 0.05); a^ǂ^ = highly significant (*p* ≤ 0.001)

Baseline vs 24 months follow-up: b = significant (*p* < 0.05); b^ǂ^ = highly significant (*p* ≤ 0.001)

5 vs 24 months follow-up: c = significant (*p* < 0.05); c^ǂ^ = highly significant (*p* ≤ 0.001)

^*^Significant difference between visits (*p* < 0.05, Friedman test or repeated measures ANOVA)

^**^Highly significant difference between visits (*p* ≤ 0.001, Friedman test or repeated measures ANOVA)

^a^Friedman test or repeated measures ANOVA when parametric test criteria were fulfilled

^b^Wilcoxon signed-rank or *t*-tests when parametric test criteria were fulfilled

Effect sizes from baseline to 5 months follow-up were ‘small’ for HADS-A and -D, ‘moderate’ for PDQ-8 SI, SCOPA-A, -B, and dopamine agonists LEDD, and ‘large’ for PDSS, SCOPA-C, and total LEDD (see Table 2). From baseline to 24 months follow-up effect sizes were ‘small’ for PDSS and SCOPA-B, ‘moderate’ for SCOPA-A, -C, and dopamine agonists LEDD, and ‘large’ for total LEDD.

**Table 2:** Relative changes and effect sizes at 5 months and 24 months follow-up

	Baseline to 5 months follow-up		Baseline to 24 months follow-up		5 to 24 months follow-up	
	RC [%]	ES^a^	RC [%]	ES^a^	RC [%]	ES^a^
PDSS total score*	23.4	0.83	9.9	0.35	− 11.0	0.53
PDSS item 1: ‘overall sleep quality’*	54.8	0.74	35.7	0.48	− 12.3	0.31
PDSS item 2: ‘sleep onset insomnia’	23.8	0.42	11.1	0.19	− 10.3	0.29
PDSS item 3: ‘sleep maintenance insomnia’**	50.0	0.65	39.6	0.51	− 6.9	0.16
PDSS item 4: ‘nocturnal restlessness in legs or arms’	38.0	0.50	8.0	0.11	− 21.7	0.44
PDSS item 5: ‘fidgeting in bed’	30.8	0.42	11.5	0.16	− 14.7	0.29
PDSS item 6: ‘distressing dreams at night’*	3.8	0.11	− 7.6	0.21	− 11.0	0.33
PDSS item 7: ‘distressing hallucinations at night’*	5.7	0.25	5.7	0.25	0.0	0.00
PDSS item 8: ‘nocturia’	2.9	0.03	0.0	0.00	− 2.9	0.03
PDSS item 9: ‘urinary incontinence during motor OFF’	11.5	0.27	− 1.3	0.03	− 11.5	0.42
PDSS item 10: ‘wakefulness due to numbness/tingling’*	14.5	0.29	11.6	0.24	− 2.5	0.07
PDSS item 11: ‘wakefulness due to painful muscle cramps’	18.5	0.36	9.2	0.18	− 7.8	0.21
PDSS item 12: ‘early waking due to painful posturing’*	22.2	0.38	15.9	0.27	− 5.2	0.13
PDSS item 13: ‘tremor on wake up’**	25.8	0.42	33.9	0.55	6.4	0.16
PDSS item 14: ‘sleep refreshment’	17.2	0.29	6.9	0.11	− 8.8	0.19
PDSS item 15: ‘unexpectedly falling asleep at daytime’*	32.2	0.51	23.7	0.38	− 6.4	0.17
PDQ-8 SI	− 31.4	0.61	− 9.1	0.18	32.6	0.52
HADS-A	− 22.7	0.36	− 8.6	0.14	18.3	0.26
HADS-D	− 20.4	0.34	6.9	0.11	35.4	0.47
SCOPA-A**	− 31.7	0.67	− 27.8	0.58	5.8	0.10
SCOPA-B	− 27.3	0.58	− 13.0	0.28	19.6	0.39
SCOPA-C**	− 47.2	0.81	− 45.3	0.77	3.6	0.04
LEDD total (mg)***	− 41.9	0.92	− 36.4	0.80	9.5	0.17
LEDD dopamine agonists (mg)**	− 47.5	0.57	− 54.5	0.65	− 13.3	0.15

*ES* effect size, *HADS-A and -D* Hospital Anxiety and Depression Scale -anxiety and -depression subscales, *LEDD* levodopa equivalent daily dose, *PDSS* Parkinson’s Disease Sleep Scale, *PDQ-8 SI* 8-item Parkinson’s Disease Questionnaire Summary Index, *RC* relative change, *SCOPA-A, -B -C* Scales for outcomes in Parkinson’s disease-motor examination, -activities of daily living, -motor complications

^*^‘small’ effect size from baseline to 24 months follow-up

^**^‘moderate’ effect size from baseline to 24 months follow-up

^***^‘large’ effect size from baseline to 24 months follow-up

^a^Effect sizes: ‘small’ (0.20–0.49), ‘moderate’ (0.50–0.79) and ‘large’ (≥ 0.80)

We recorded psychotropic medication in all patients: stable treatment regimens from baseline to last assessment were administered for two patients with quetiapine (25 and 50 mg), one patient with agomelatine (25 mg), one patient with amitriptyline (100 mg), one patient with opipramole (50 mg), and one patient with citalopram (50 mg). In two patients psychotropic medication changed during the course of the study: one patient was switched from mirtazapine (30 mg) to quetiapine (100 mg) at 5 months follow-up as visual hallucinations had developed and one patient was postoperatively started on quetiapine (150 mg) as the patient developed suicidal ideation.

### Explorative analyses of PDSS items at baseline, 5 months, and 24 months follow-up

Friedman-tests for PDSS items found significant longitudinal changes of all items except ‘Nocturia’ (see Table 1).

Comparing baseline to 5 months follow-up, post-hoc Wilcoxon tests resulted in significant improvements of ‘overall sleep quality’ (*p* < 0.001), ‘sleep onset insomnia’ (*p* = 0.003), ‘sleep maintenance insomnia’ (*p* < 0.001), ‘nocturnal restlessness in legs or arms’ (*p* < 0.001), ‘fidgeting in bed’ (*p* = 0.001), ‘distressing hallucinations at night’ (*p* = 0.006), ‘urinary incontinence during motor OFF’ (*p* = 0.020), ‘wakefulness due to numbness/tingling’ (*p* = 0.012), ‘wakefulness due to painful muscle cramps’ (*p* = 0.004), ‘early waking due to painful posturing’ (*p* = 0.001), ‘tremor on wake up’ (*p* = 0.002), ‘sleep refreshment’ (*p* = 0.029), and ‘unexpectedly falling asleep at daytime’ (*p* < 0.001).

Comparing baseline to 24 months follow-up, post-hoc Wilcoxon tests resulted in significant improvements of ‘overall sleep quality’ (*p* < 0.001), ‘sleep maintenance insomnia’ (*p* = 0.001), ‘early waking due to painful posturing’ (*p* = 0.014), ‘tremor on wake up’ (*p* < 0.001), and ‘unexpectedly falling asleep at daytime’ (*p* = 0.003). In contrast, a significant worsening was observed for ‘distressing dreams at night’ (*p* = 0.030). No significant changes were found for other PDSS items.

Effect sizes of improvements from baseline to 24 months follow-up were ‘small’ for ‘overall sleep quality’, ‘wakefulness due to numbness/tingling’, ‘early waking due to painful posturing’, ‘unexpectedly falling asleep at daytime’, and ‘moderate’ for, ‘sleep maintenance insomnia’ and ‘tremor on wake up’ (see Table 2). For PDSS items with worsening scores at 24 months follow-up, ‘small’ effect sizes were found for ‘distressing dreams at night’ and ‘distressing hallucinations at night’. Other effect sizes were negligible.

### Explorative correlation analyses between outcome parameters at 5 months and 24 months follow-up

PDSS total score improvement significantly correlated with improvements of PDQ-8 SI and SCOPA-C at 24 months follow-up (see Table 3). No significant correlations were found for improvements of SCOPA-A and -B, HADS-A and -D, and LEDD reduction (total and dopaminagonists). Explorative analyses of change scores (1) from baseline to 24 months follow-up and (2) from 5 to 24 months follow-up resulted in no significant correlations between LEDD (total and dopamine agonists) and PDSS items).

**Table 3:** Spearman correlations between outcomes at 24 months follow-up

		PDSS	PDQ-8 Summary Index	HADS-A	HADS-D	SCOPA-A	SCOPA-B	SCOPA-C
PDQ-8 SI	rho	− 0.322**						
	*p*	**0.007**						
	*n*	70						
HADS-A	rho	− 0.161	0.444**					
	*p*	0.191	** < 0.001**					
	*n*	68	71					
HADS-D	rho	− 0.100	0.318**	0.561**				
	*p*	0.418	**0.007**	** < 0.001**				
	*n*	68	71	71				
SCOPA-A	rho	− 0.042	.239*	0.071	0.086			
	*p*	0.741	**0.050**	0.571	0.491			
	*n*	65	68	67	67			
SCOPA-B	rho	− 0.140	0.311**	0.144	0.295*	0.570**		
	*p*	0.251	**0.008**	0.234	**0.013**	** < 0.001**		
	*n*	69	72	70	70	68		
SCOPA-C	rho	− .341**	0.298*	0.266*	0.089	− 0.008	0.195	
	*p*	**0.004**	**0.011**	**0.026**	0.462	0.951	0.101	
	*n*	69	72	70	70	68	72	
LEDD total	rho	− 0.006	− 0.092	− 0.116	0.036	− 0.145	− 0.191	− 0.270*
	*p*	0.963	0.446	0.342	0.767	0.247	0.113	**0.024**
	*n*	68	71	69	69	66	70	70
LEDD dopamine -agonists	rho	− 0.056	0.084	0.188	0.161	− 0.016	− 0.207	− 0.147
	*p*	0.644	0.478	0.117	0.179	0.894	0.081	0.219
	*n*	68	71	69	69	66	70	70

Bold font highlights significant results

Higher PDSS total scores indicate less sleep–wake disturbances. Higher test PDQ-8 SI, HADS-A and -D, SCOPA-A, -B, and -C indicate more impairment of specific symptoms. Therefore, significant correlations with negative correlation coefficients between PDSS total and PDQ-8 SI and SCOPA-C indicate that an improvement of sleep is associated with improvements of QoL and motor complications

*HADS-A and –D* Hospital Anxiety and Depression Scale-anxiety and -depression subscales, *LEDD* levodopa equivalent daily dose, *PDSS* Parkinson’s Disease Sleep Scale, *PDQ-8 SI* 8-item Parkinson’s Disease Questionnaire Summary Index, *rho* Spearman’s correlation coefficient, *SCOPA-A, -B and –C* Scales for outcomes in Parkinson’s Disease-motor examination, -activities of daily living, and -motor complications

^*^Significant correlation at the 0.05 level (2-tailed)

^**^Significant correlation at the 0.01 level (2-tailed)

## Discussion

In this prospective, observational, international, multicenter study including 73 patients with PD, we observed significant beneficial effects of bilateral STN-DBS on QoL, sleep and motor symptoms at 5 months and 24 months follow-up.

### Effects of STN-DBS on specific aspects of sleep

The following specific aspects of sleep in PD significantly changed in the longitudinal follow-up of the study:

- Sleep onset and maintenance insomnia (items 2 and 3): in line with previous studies, we observed an improvement of both types of insomnia at short-term follow-up [25]. Studies using polysomnography support these findings with evidence of improvements of sleep continuity and depth [3], total sleep time and efficiency [1], and wakefulness after sleep onset which correlated with an improvement of the PDSS total score [37].
- Nocturnal restlessness (items 4 and 5): in line with previous studies, we observed an improvement of nocturnal restlessness at short term-follow-up [3, 4, 25].
- Nocturnal psychosis (items 6 and 7): previous studies have reported conflicting results for nocturnal psychosis. A study by Peppe et al. including five patients with PD reported a significant improvement of ‘distressing dreams at night’ at short-term follow-up in pedunculopontine DBS [39]. In contrast, a study by Hjort et al. including ten patients with PD undergoing STN-DBS found no evidence for an improvement of this aspect [25] which was confirmed by the results in our cohort. As neither study reported an improvement of ‘distressing hallucinations at night’, the present study is the first one to report a beneficial effect of STN-DBS on this aspect at short-term follow-up. A connection to changes in dopaminergic medication seems possible, as the postoperative total LEDD reduction was > 40% at 5 months and > 35% at 24 months follow-up. However, correlation analyses provide no evidence for a linear relationship between improvements of total or dopamine agonists LEDD reduction and an improvement of specific PDSS items, such as nocturnal psychosis. Further studies are needed to investigate this issue.
- Nocturnal urinary symptoms (items 8 and 9): confirming negative results from previous studies [4, 6, 25], we found no effects of STN-DBS on ‘Nocturia’. However, to our knowledge, the present study is the first one to report an improvement of ‘urinary incontinence during motor OFF’ at 5 months follow-up. This observation could be explained by an improvement of nocturnal motor symptoms.
- Nocturnal sensorimotor symptoms (PDSS items 10–13): In line with previous studies, we found beneficial effects of bilateral STN-DBS on all PDSS items for nocturnal sensorimotor symptoms [25] at short-term 5 months follow-up.
- Sleep refreshment (item 14): confirming previous studies we found a significant improvement of sleep refreshment at short-term follow-up [4]. Contrary to the results published by Choi et al., we observed a significant beneficial effect on sleep refreshment at 24 months follow-up.
- Daytime sleepiness (item 15): previous studies have reported negative results for this aspect of sleep–wake disturbances at short-term [25] and long-term [6, 30] follow-up after STN-DBS. To our knowledge, the present study is the first one to report significant beneficial effects of STN-DBS on daytime sleepiness at long-term follow-up. Although a link between dopaminergic medication, in particular dopamine agonists, and daytime sleepiness or sleep attacks is well known [26], we found no evidence for a linear relationship between a total or dopamine agonists LEDD reduction and an improvement of this PDSS item. Further studies are needed to investigate possible higher-order relationships which might result from patient-specific adverse events thresholds.
- Overall sleep quality (item 1): in line with previous studies, the overall quality of sleep significantly improved at short-term [4, 25] and 24 months long-term follow-up [6]. This may be a result of the above mentioned improvements of specific PDSS domains.

### Mechanisms of effects of STN-DBS on sleep

Sleep–wake disturbances are a collection of different symptoms and result from multi-neuropeptide dysfunction including the central dopaminergic, hypocretinergic, noradrenergic, and serotonergic systems [21]. As the pathomechanisms of sleep–wake disturbances are diverse, various mechanisms of action may influence the effects of STN-DBS [10]:

- A direct modulation of the basal ganglia-thalamo-cortico loops may influence neural activity, e.g., in the motor circuitry which in turn could improve motor symptoms-related sleep disorder [29]. Furthermore, a modulation of the medial thalamus could, e.g., improve restlessness in legs or arms [19]. Future studies are required to assess the role of directional DBS towards subregions of the STN and the adjacent target region [16, 17, 40]. Another possible explanation could be mediated through projections from the STN to the globus pallidus externus as electrophysiological animal studies have shown that during STN-DBS the activity in the globus pallidus externus is increased which may result in an improvement of sleep [22, 24, 27, 34, 41].
- A spread of current to nuclei in proximity of the STN, such as the pedunculopontine nucleus, which has previously been associated with an improvement of nighttime sleep and daytime sleepiness [39, 43]. While the exact borders of the pedunculopontine nucleus are difficult to define [23], a location approximately 5 mm ventral of the STN with even closer projections has been discussed [36].
- The reduction of dopaminergic medication requirements below patient-specific thresholds may at least in part influence sleep–wake disturbances, such as daytime sleepiness [26] or hallucinations [48]. Further studies are needed to distinguish between stimulation and medication effects on these NMS.

### Relationship of sleep and other outcome parameters

The significant correlation between improvements of PDSS total score and PDQ-8 SI indicates the close connection between sleep and QoL outcomes. The fact that Spearman correlations showed a significant relationship between improvements of motor complications and sleep symptoms is consistent with the observation that nocturnal motor symptoms, such as painful dystonic posturing, were improved at 24 months-follow-up and indicate the relative importance of nocturnal motor symptoms for subjective sleep outcomes. The observation that improvements of PDSS total score and HADS-A and -D were not significantly correlated indicates that sleep and mood disorders are separately influenced by STN-DBS. Psychotropic medication was started or switched only in 2 out of 73 patients of our cohort during the course of this study. Therefore, it seems unlikely that observed beneficial effects of STN-DBS on sleep symptoms were based on these drugs. Additionally, as discussed above no relationship was found between changes of sleep symptoms and changes of dopaminergic and specifically dopamine agonist medication. As also the medication changes from 5 to 24 months follow-up were not correlated with PDSS changes, one might argue that medication side effects are unlikely causes for the observed changes of sleep symptoms between follow-up visits and therefore factors like disease progression or chronic DBS treatment itself might also contribute to the observed changes. Further studies are needed to investigate this issue.

### Limitations

A number of limitations should be considered in this study. Although the cohort size in our study (*n* = 73) is one of the biggest in studies of its kind, the study cohort is relatively small and further prospective studies are required to confirm these findings. The multicenter design of our study is likely to reduce systematic bias caused by single center studies. We did not include laboratory-assisted assessments of sleep, such as multiple sleep latency test or polysomnography for sleep architecture as these measures require additional equipment and are rather costly. However, we were interested in a pragmatic assessment of a wide range of sleep–wake disturbances including complex symptoms, such as nocturia, nocturnal psychosis, motor state-related sleep symptoms, and sleep refreshment, which cannot be captured by polysomnography. Due to the design of our database as a prospective, observational study, motor assessments were recorded in ON states (MedON/StimON) [15]. Although the current study did not find a relationship/correlation between change in motor exam and change in sleep, this is still an important potential contributor to the improvement in sleep and the relationship may have been masked because participants were only evaluated in ON states. Many of the studies that did find a relationship between motor and sleep outcomes used polysomnography. Furthermore, systematic follow-up examinations of motor states with and without medication and DBS could also provide useful information on patients’ non-dopaminergic, non-motor characteristics which may contribute to their sleep–wake disturbances [21]. Furthermore, this study did not assess apathy in detail and analyze the interplay between apathy and sleep/fatigue observed in previous studies by Eugster et al. and Bargiotas et al. [2, 20].

## Conclusion

We observed significant long-term beneficial effects of STN-DBS on overall quality of sleep and a wide range of specific sleep symptoms, such as sleep maintenance insomnia, early waking due to painful posturing, tremor on wake up, and daytime sleepiness. Improvements of sleep symptoms seem to be, at least in part, mediated by nocturnal motor symptoms. A significant correlation between sleep and QoL outcomes at 24 months follow-up epitomizes the relative importance of sleep symptoms for the holistic assessment of DBS outcomes.

## Data Availability

The data included in this study is available on request to the corresponding author. The data are not publicly available due to their containing information that could compromise the privacy of the participants.

## Abbreviations

DBS: Deep brain stimulation
LEDD: Levodopa equivalent daily dose
PDSS: Parkinson’s Disease Sleep Scale
PDQ-8 SI: 8-Item PD Questionnaire Summary Index
QoL: Quality of life
SCOPA-A, -B, and -C: Scales for outcomes in PD-motor examination, activities of daily living, and motor complications
STN: Subthalamic nucleus
